# Retinal microvasculature dysfunction is associated with Alzheimer’s disease and mild cognitive impairment

**DOI:** 10.1186/s13195-020-00724-0

**Published:** 2020-12-04

**Authors:** Jacqueline Chua, Qinglan Hu, Mengyuan Ke, Bingyao Tan, Jimmy Hong, Xinwen Yao, Saima Hilal, Narayanaswamy Venketasubramanian, Gerhard Garhöfer, Carol Y. Cheung, Tien Yin Wong, Christopher Li-Hsian Chen, Leopold Schmetterer

**Affiliations:** 1 Singapore Eye Research Institute, Singapore National Eye Centre, 20 College Road, The Academia, Level 6, Discovery Tower, Singapore, 169856 Singapore; 2 Ophthalmology and Visual Sciences Academic Clinical Program, Duke-NUS Medical School, National University of Singapore, Sha Tin, Singapore; 3 SERI-NTU Advanced Ocular Engineering (STANCE), Sha Tin, Singapore; 4 Institute for Health Technologies, Nanyang Technological University, Sha Tin, Singapore; 5 Memory Aging and Cognition Centre, Departments of Pharmacology and Psychological Medicine, Yong Loo Lin School of Medicine, National University of Singapore, Sha Tin, Singapore; 6 Saw Swee Hock School of Public Health, National University of Singapore, Sha Tin, Singapore; 7 Department of Pharmacology, National University of Singapore, Singapore, Singapore; 8 Raffles Neuroscience Centre, Raffles Hospital, Singapore, Singapore; 9 Department of Clinical Pharmacology, Medical University Vienna, Vienna, Austria; 10 Department of Ophthalmology and Visual Sciences, The Chinese University of Hong Kong, Sha Tin, Hong Kong; 11 Center for Medical Physics and Biomedical Engineering, Medical University Vienna, Vienna, Austria; 12 Institute of Molecular and Clinical Ophthalmology, Basel, Switzerland

**Keywords:** Optical coherence tomography angiography, Alzheimer’s disease, Mild cognitive impairment

## Abstract

**Background:**

The retina and brain share many neuronal and vasculature characteristics. We investigated the retinal microvasculature in Alzheimer’s disease (AD) and mild cognitive impairment (MCI) using optical coherence tomography angiography (OCTA).

**Methods:**

In this cross-sectional study, 24 AD participants, 37 MCI participants, and 29 controls were diagnosed according to internationally accepted criteria. OCTA images of the superficial and deep capillary plexus (SCP, DCP) of the retinal microvasculature were obtained using a commercial OCTA system (Zeiss Cirrus HD-5000 with AngioPlex, Carl Zeiss Meditec, Dublin, CA). The main outcome measures were vessel density (VD) and fractal dimension (FD) in the SCP and DCP within a 2.5-mm ring around the fovea which were compared between groups. Perfusion density of large vessels and foveal avascular zone (FAZ) area were additional outcome parameters.

**Results:**

Age, gender, and race did not differ among groups. However, there was a significant difference in diabetes status (*P* = 0.039) and systolic blood pressure (*P* = 0.008) among the groups. After adjusting for confounders, AD participants showed significantly decreased VD in SCP and DCP (*P* = 0.006 and *P* = 0.015, respectively) and decreased FD in SCP (*P* = 0.006), compared to controls. MCI participants showed significantly decreased VD and FD only in SCP (*P* = 0.006 and *P* < 0.001, respectively) and not the DCP (*P* > 0.05) compared with controls. There was no difference in the OCTA variables between AD and MCI (*P* > 0.05). Perfusion density of large vessels and FAZ area did not differ significantly between groups (*P* > 0.05).

**Conclusions and relevance:**

Eyes of patients with AD have significantly reduced macular VD in both plexuses whereas MCI participants only showed reduction in the superficial plexus. Changes in the retinal microvasculature and capillary network may offer a valuable insight on the brain in AD.

**Supplementary information:**

The online version contains supplementary material available at 10.1186/s13195-020-00724-0.

## Introduction

Alzheimer’s disease (AD) is a significant cause of dementia and has important implications for patients and their families. Globally, the number of individuals living with dementia is set to rise, particularly in low- and middle-income countries [1]. The retina and brain share many neuronal and vasculature characteristics [2], and potential biomarkers may be present in the retina. Previous studies have analyzed digital fundus photographs and reported a range of retinal vessel alterations in patients with AD and mild cognitive impairment (MCI) [3]. However, images obtained from this technique can only provide information of retinal arterioles and venules measuring 60–300 μm in diameter [4]. Optical coherence tomography angiography (OCTA) is a recent innovation that allows for further quantification of the retinal microvasculature and visualization of capillaries measuring 5–15 μm in diameter, which may be more representative of the entire microvascular network [5, 6]. Thus, the OCTA may be a potential non-invasive optical imaging tool to determine the presence and role of microvascular dysfunction in AD and cognitive impairment.

Furthermore, OCTA is capable of measuring retinal capillary beds at distinct depths, separating the superficial capillary plexuses (SCP) and deep capillary plexuses (DCP), each reflecting the metabolic demand of particular neuronal layers [5]. In AD, the tissue of interest is the inner retinal layer, as suggested by changes in retinal ganglion cells [7, 8], thinning of the retinal nerve fiber layer thickness and ganglion cell layer thickness [9], and deposition of β-amyloid (Aβ) plaques [10]. It is important to highlight that these retinal changes in AD will require confirmation in studies with comparable methods in patient selection, scanning protocols, and analyses.

While there are a few OCTA studies investigating AD, there have been mixed conclusions [11–15]. Some researchers reported finding significant reduction in the vessel density (VD) only in the superficial plexus [14, 16], which complements histology findings [10] and OCT studies [9] since the superficial plexus mainly supplies the inner retinal layer [17]. However, others reported finding changes only in the deep plexus [11, 15]. Studies [15, 16] have also used OCTA to examine participants with MCI, who are at higher risk for dementia and AD, but have drawn conflicting results as well. For example, while Zhang et al. [16] found significantly decreased VD only in the superficial plexus, Wu et al. [15] found a reduction in the VD only in the deep plexus. Therefore, it remains unclear whether there is a difference in the OCTA parameters in AD and MCI individuals, partly due to the physiologic variability of the foveal avascular zone (FAZ) [18, 19], and projection artifacts of the DCP [20, 21].

To address these gaps, the purpose of the current study is to compare the retinal microvasculature metrics using OCTA, accounting for potential measurement bias of FAZ and projection artifacts of DCP in participants with AD, MCI, and controls. We hypothesize that alterations in OCTA metrics as characterized by sparser vessel density and loss of vessel complexity will occur predominately within the superficial capillary plexus, in AD and to a lesser extent in MCI compared to controls. We would also like to explore if other retinal parameters such as the perfusion density of large retinal vessels and area of FAZ can provide meaningful findings between individuals who are cognitively impaired and controls.

## Methods

### Study participants

We conducted a cross-sectional case-control study, approved by National Healthcare Group Pte Ltd. Domain Specific Review Board and adhered to the Declaration of Helsinki. Written informed consent was obtained from each participant or their primary caregiver before enrollment.

#### AD and MCI individuals

Participants aged 50 years of age or older were enrolled from an ongoing longitudinal memory clinic-based study from December 2018 to October 2019. Weekly consensus meetings were held with study clinicians, neuropsychologists, clinical research fellows, research coordinators, and research assistants. Details from the clinical assessment, blood investigations, neuropsychological testing, and MRI scans were reviewed. AD participants were diagnosed in accordance with the Diagnostic and Statistical Manual of Mental Disorders (DSM)-IV criteria [22]. MCI participants were those who were defined as impairment in 1 or more domains in the neuropsychological test battery that does not affect activities of daily living.

#### Controls

These are individuals who attended the same clinics but were not impaired in any of the cognitive domains tested. Medical histories (e.g., diabetes and hypertension) were collected, and seated blood pressure (BP) measurements were taken using an automated oscillometric device during their clinical visits. Participants were excluded from this study if they were hypoxic, anoxic, hypotensive, hypertensive, uremic, or hepatic encephalopathy; had traumatic, nutritional, or toxic disorder affecting the central nervous system (CNS); had any current or past substance abuse disorder that has affected the CNS; and had intracerebral hemorrhage, cranial arteritis, CNS inflammatory vasculitis, Moyamoya disease, CNS infection, space occupying intracranial mass lesion, obstructive or normal pressure hydrocephalus, difficulty in controlling epilepsy, medical illness requiring concomitant corticosteroid or immunosuppressant therapy, moribund state, significant aphasia, or dysarthria that will significantly impede cognitive assessment.

### Neuropsychological and neuroimaging protocols

All participants received the Mini-Mental State Examination (MMSE), which is a brief dementia screening instrument with a total score of 30, and a higher score indicates better cognition [23]. MRI scans were performed on a 3-T Siemens Magnetom Trio Tim scanner (Siemens AG, Medical Solutions, Germany), using a 32-channel head coil at Clinical Imaging Research Center, National University of Singapore. MRI scans were graded for markers of vascular injury following the previously described procedures [24]. Briefly, medial temporal lobe atrophy (MTA) was graded on the Scheltens Scale [25] and global cortical atrophy (GCA) was graded on the Global Cortical Atrophy Scale [26].

MTA scores were graded from 0 to 4, where 0 = no, 1 = mild, 2 = mild to moderate, 3 = moderate, and 4 = severe. GCA scores were graded from 0 to 3, where 0 = no, 1 = mild, 2 = moderate, and 3 = severe. White matter hyperintensities (WMH) were graded using the Modified Fazekas Scale and recorded as 0 = no, 1 = punctate, 2 = beginning, and 3 = confluent areas [27].

### Ocular examinations

After pupil dilation with 1% tropicamide and 2.5% phenylephrine hydrochloride, each participant underwent ocular imaging in both eyes that included retinal photography with a nonmydriatic digital camera and OCTA imaging. Two retinal fundus photographs, with one centered at the optic disc and another centered at the macula, were obtained to document the absence of eye diseases.

### Optical coherence tomography angiography

All OCTA scans were performed by a single trained technician, using the Zeiss Cirrus HD-5000 Spectral-Domain OCT with AngioPlex OCTA (Carl Zeiss Meditec, Dublin, CA) that featured a central wavelength of 840 nm, a speed of 68,000 A-scan per second, and an axial resolution of 5 μm and transverse resolution of 15 μm in tissue. The FastTrac motion correction, based on a line-scanning ophthalmoscope, was enabled to minimize motion artifacts during acquisition. Each participant received a 3 × 3-mm^2^ scan, with each scan consisting of an isotropic sampling (245 × 245) and four consecutive B-scans obtained at each raster location to compute the angiographic information using an optical microangiography protocol [28].

A trained grader masked to the participant’s characteristics reviewed the quality of all OCTA scans. All B-scans were checked for alignment and segmentation errors. We excluded participants from the analysis if the OCTA images from both eyes were of poor quality (poor signal strength index < 7, significant motion artifacts visible as irregular vessel patterns on the en face angiogram, local weak signal caused by artifacts such as floaters, misalignment or incorrect segmentation) [29, 30]. A randomly selected eye was analyzed for each participant since measurements of both eyes were highly correlated.

Each scan was automatically segmented into the superficial capillary plexus (SCP) and deep capillary plexus (DCP) by a review software (Carl Zeiss Meditec, version 11.0.0.29946). The SCP spans the inner limiting membrane (ILM) to the inner plexiform layer (IPL), while the DCP spans the inner nuclear layer (INL) to the outer plexiform layer (OPL) [31]. Images were checked to ensure correct segmentation by the automated instrument software, and no manual adjustment was needed. Projection artifacts from the overlying retinal circulation were removed from the DCP using the removal software that was integrated with the instrument.

**Fig. 1:**
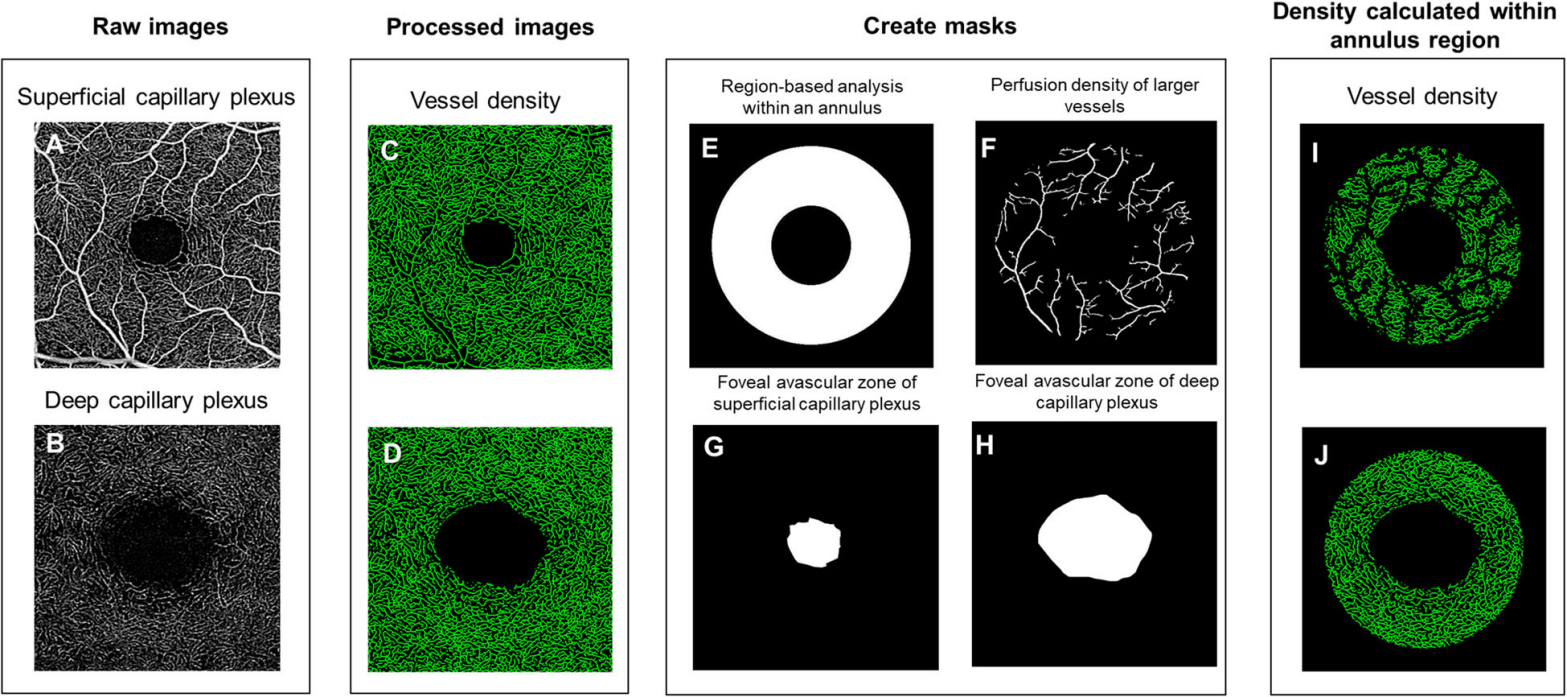
The framework of optical coherence tomography angiography (OCTA) image post-processing. **a**, **b** Raw images were extracted from the OCTA machines. **c**, **d** The images were binarized to obtain the large vessel densities. **e** An annulus centered at FAZ center with inner diameter of 1 mm and outer diameter 2.5 mm was generated as a mask to the 3 × 3 mm image. **f** Larger vessels were automatically detected in the superficial capillary plexus. **g**, **h** The FAZs were manually delineated from both plexuses. **i**, **j** The vessel densities were calculated in the annulus

OCTA images of the superficial and deep retinal plexuses were loaded into a customized algorithm using MATLAB (The MathWorks Inc., Natick, MA). The framework of OCTA image processing involved the following steps (Fig. 1): (1) manually outlined the border of the foveal avascular zone (FAZ) of the superficial and deep vascular plexus angiograms [32]; (2) applied a Hessian-based filter to enhance the contrast of large vessels on the SCP, which is consequently (3) binarized [33, 34]; and (4) performed the region-based analysis with a fovea-centered annulus that has an inner diameter of 1 mm and outer diameter of 2.5 mm. (5) VD was defined as the total length in millimeter of perfused retinal microvasculature per unit area in square millimeter in the annulus region of measurement. Perfusion density of the large vessels was computed as the ratio between large vessel area per total imaged area in the annulus region of measurement. (6) FD represents the vessel complexity of the retinal vasculature [35] and was calculated within the annulus zone using the box counting method (Dbox) with the fractal analysis toolbox (TruSoft Benoit Pro 2.0, TruSoft International, Inc., St. Petersburg, FL) [36].

### Statistical analyses

We did a post hoc power calculation to evaluate the statistical power of current existing study (*n* = 24 AD cases vs 29 controls) using the means and standard deviations derived from Table 4. For superficial capillary plexus (30.54 + 2.86% vs 32.60 + 2.36%) [12–14, 16, 37], using an alpha error of 5%, we would have a post hoc power of 80.5%. For the deep capillary plexus, using 43.5 + 4.0% vs 50.9 + 3.6% [11, 15], we would have a post hoc power of 100% (https://clincalc.com/stats/Power.aspx) [38].

To compare the characteristics of participants among groups, one-way analysis of variance (ANOVA) was performed for continuous variables and chi-square tests were performed for categorical variables. Associations between systemic factors (independent variables) with OCTA variables (dependent variable) were assessed using multivariable linear regression analysis. We adjusted for age, gender, race, and included factors with biologically plausible relations to cognitive status and/or OCTA variable, such as the presence of diabetes, blood pressure levels (systolic and diastolic), and signal strength of OCTA scans [29, 30, 37, 39]. The primary outcome of the study was VD and FD, and the secondary outcome was perfusion density of large retinal vessels and FAZ area. To avoid α error accumulation due to multiple testing, we used a conservative Bonferroni correction and considered results statistically significant at the level α = 0.05/2 = 0.025 for primary and secondary outcomes. For all other analyses, a *P* value < 0.05 was considered statistically significant. The diagnostic accuracy of the OCTA parameter (in terms of statistical significance and effect measure) to differentiate between control and AD/MCI participants was compared by means of the area under the receiver operating characteristic area under the curve (AUC). Statistical analyses were performed using Stata version 16.0 (StataCorp LLC, College Station, TX).

## Results

Additional file 1 (Figure S1) detailed the inclusion and exclusion criteria of the study participants. Of the 165 participants who were enrolled and imaged between December 2018 and October 2019, we excluded participants who were unable to complete OCTA scanning due to fatigue (*n* = 15), poor scan quality (*n* = 53), and presence of eye diseases such as glaucoma, vascular or nonvascular retinopathies, and age-related macular degeneration which were ascertained from both fundus photographs and OCTA scans (*n* = 7), leaving 24 AD participants, 37 MCI participants, and 29 control participants with good quality OCTA for analysis. There was no difference in terms of the scan quality (*P* = 0.133) and eye disease status (*P* = 0.476) among the groups.

There was no significant difference in age, gender, and race, among the groups (Table 1). The mean ± standard deviation (SD) age of participants was 76.8 ± 6.0 years, 51% were female, and 81% were Chinese. Of note, AD participants had higher diabetes prevalence (*P* = 0.039), higher levels of systolic BP (*P* = 0.008), lower MMSE scores, and higher GCA scores (*P* = 0.006) than participants with MCI or controls. There was a small but significant difference in the average OCTA quality in participants with AD (9.8 ± 0.8 signal strength), MCI participants (9.8 ± 0.5 signal strength), and controls (9.6 ± 0.7 signal strength; *P* = 0.010). MTA and WMH data did not show differences by cognitive status (*P* = 0.098).

**Table 1:** Characteristics of participants by cognitive status

Characteristics	AD (***n*** = 24)	MCI (***n*** = 37)	Control (***n*** = 29)	***P*** value*
Age	74.9 ± 6.0	77.9 ± 6.4	76.7 ± 5.3	0.176
Gender, female	17 (73)	16 (44)	13 (45)	0.082
Race, Chinese	16 (84)	30 (82)	23 (79)	0.082
Diabetes, yes	9 (41)	10 (32)	4 (14)	**0.039**
Hypertension, yes	15 (78)	21 (62)	17 (59)	0.319
Systolic blood pressure, mmHg	148 ± 11	139 ± 18	131 ± 16	**0.008**
Diastolic blood pressure, mmHg	73 ± 9	70 ± 9	70 ± 8	0.363
MMSE	20.3 ± 6.1	23.9 ± 6.3	24.8 ± 4.8	**0.026**
Neuroimaging markers				
MTA scores	1.3 ± 0.7	1.4 ± 0.8	1.7 ± 0.7	0.297
GCA scores	1.5 ± 0.6	1.6 ± 0.6	2.3 ± 0.8	**0.006**
WMH scores	1.6 ± 0.7	1.5 ± 0.7	2.1 ± 0.7	0.098
Signal strength, out of 10	9.8 ± 0.8	9.8 ± 0.5	9.6 ± 0.7	**0.010**

Data presented are mean (SD) or number (%), as appropriate

*AD* Alzheimer’s disease, *MCI* mild cognitive impairment, *MMSE* Mini-Mental State Exam, *MTA* medial temporal atrophy, *GCA* global cortical atrophy, *WMH* white matter hyperintensities

*P value was obtained with ANOVA for the continuous variables and with chi-square tests for categorical variables

After adjusting for age, gender, race, diabetes, and blood pressure (systolic and diastolic levels), VD in the SCP of the AD, MCI, and control groups was 14.78 ± 1.14, 14.94 ± 1.02, and 15.66 ± 0.96, respectively. A significant difference of VD in the SCP was noted between the control group and the AD and MCI (*P* = 0.006); however, no significant difference was noted between the AD and MCI groups. The VD in the DCP of the AD and control groups was significantly different (20.42 ± 1.60 and 21.54 ± 1.55, respectively; *P* = 0.015) (Table 2).

**Table 2:** Multivariate analysis of vessel density and fractal dimension with cognitive impairment

Cognitive status	Superficial capillary plexus				Deep capillary plexus			
	Mean ± SD	β	95 CI	***P*** value*	Mean ± SD	β	95 CI	***P*** value*
**Vessel density (%)**								
Control	15.66 ± 0.96	Reference			21.54 ± 1.55	Reference		
MCI	14.94 ± 1.02	− 0.72	− 1.22 to − 0.21	**0.006**	20.81 ± 1.65	− 0.73	− 1.54 to 0.09	0.081
AD	14.78 ± 1.14	− 0.88	− 1.49 to − 0.26	**0.006**	20.42 ± 1.60	− 1.12	− 2.00 to − 0.21	**0.015**
**Fractal dimension**								
Control	1.861 ± 0.010	Reference			1.879 ± 0.013	Reference		
MCI	1.850 ± 0.011	− 0.011	− 0.016 to − 0.006	**< 0.001**	1.876 ± 0.014	− 0.003	− 0.010 to 0.004	0.386
AD	1.853 ± 0.011	− 0.008	− 0.014 to − 0.002	**0.006**	1.876 ± 0.014	− 0.004	− 0.011 to 0.004	0.371

*AD* Alzheimer’s disease, *CI* confidence intervals, *MCI* mild cognitive impairment, *SD* standard deviation

*Adjusted for age, gender, race, diabetes, blood pressure (systolic and diastolic levels), and signal strength of OCTA scans

**Fig. 2:**
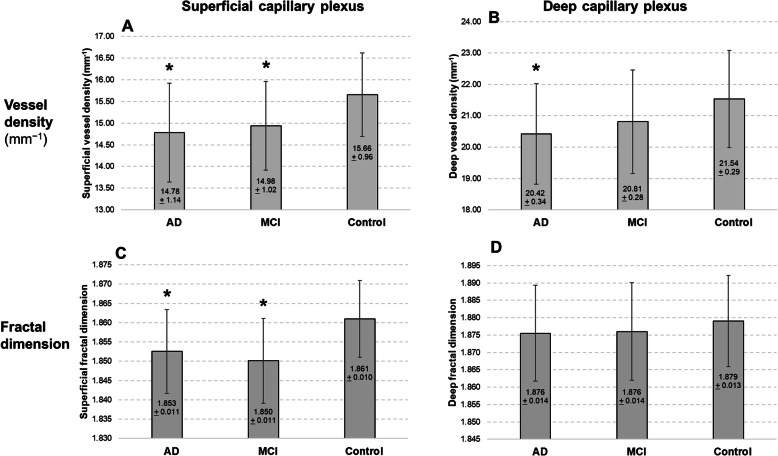
Distribution of **a** superficial vessel density, **b** deep vessel density, **c** superficial fractal dimension, and **d** deep fractal dimension stratified by participants having with Alzheimer’s disease (AD), mild cognitive impairment (MCI), and controls. Data and *P* values shown are after adjustment for age, gender, race, diabetes, and blood pressure (systolic and diastolic levels). The asterisk symbol (*) indicates a statistical significance of *P* < 0.05 when compared to the controls

**Fig. 3:**
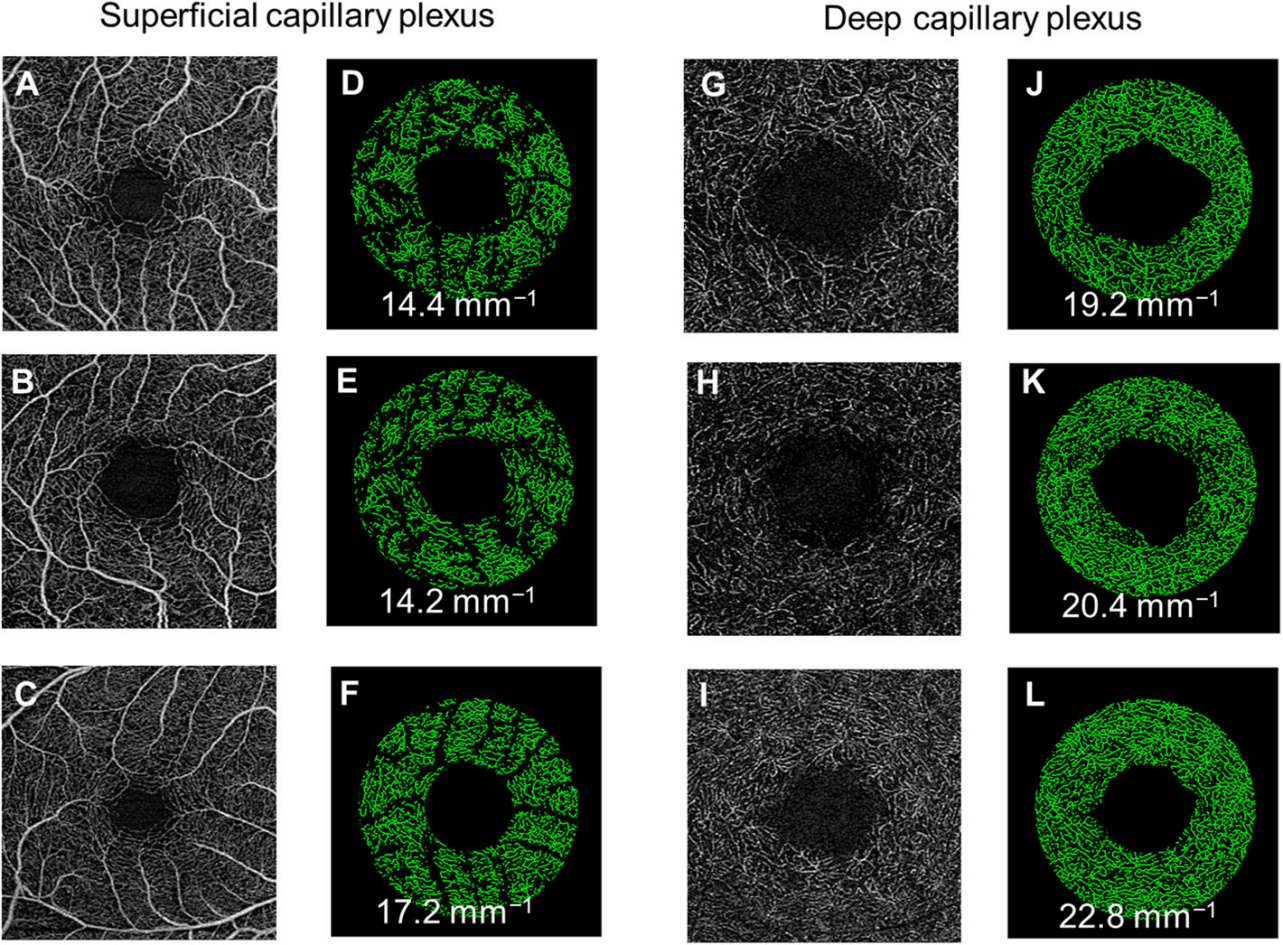
Optical coherence tomography angiography (OCTA) images of the superficial (**a**–**c**) and deep (**g**–**i**) capillary plexuses were extracted from the OCTA machines. **d**–**f**, **j**–**l** Vessel density maps of the macular annulus region showing retinal microvasculature of participants with Alzheimer’s disease (AD; **d**, **j**), mild cognitive impairment (MCI; **e**, **k**), and controls (**f**, **l**). AD participants showed a decrease in vessel densities in both plexuses compared to controls. MCI participants showed a decrease vessel density only in superficial capillary plexus and not the deep capillary plexus

In the multivariate analysis, FD in the SCP of the AD, MCI, and control groups was 1.853 ± 0.011, 1.850 ± 0.011, and 1.861 ± 0.010, respectively. A significant difference of FD in the SCP was noted between the control group and the AD (*P* < 0.001) and MCI (*P* = 0.006); however, no significant difference was noted between the AD and MCI groups. FD in the DCP did not differ between groups (*P* = 0.371). Figures 2 and 3 further illustrate the graphical representation and OCTA images of VD and FD among the groups, respectively. There were no statistically significant differences in the OCTA variables between AD and MCI (*P* > 0.05). Analysis with and without adjusting for BP levels did not alter the MCI/AD disease effects. Perfusion density of large vessels and FAZ area did not differ significantly between groups (*P* > 0.05; Table 3).

**Table 3:** Multivariate analysis of perfusion density of large vessels and foveal avascular zone with cognitive impairment

Cognitive status	Superficial capillary plexus				Deep capillary plexus			
	Mean ± SD	β	95 CI	***P*** value*	Mean ± SD	β	95 CI	***P*** value*
**Perfusion density of large vessels (%)**								
Control	6.92 ± 1.16	Reference			–		–	
MCI	6.39 ± 1.27	− 0.53	− 1.15 to 0.09	0.091	–	–	–	–
AD	7.02 ± 1.29	0.10	− 0.61 to 0.81	0.787	–	–	–	–
**Foveal avascular zone area (mm**^**2**^**)**								
Control	0.31 ± 0.12	Reference			1.11 ± 0.47	Reference		
MCI	0.35 ± 0.12	0.041	− 0.02 to 0.10	0.190	1.24 ± 0.39	0.129	− 0.07 to 0.33	0.197
AD	0.34 ± 0.14	0.034	− 0.04 to 0.11	0.380	1.13 ± 0.43	0.016	− 0.23 to 0.26	0.898

*AD* Alzheimer’s disease, *CI* confidence intervals, *MCI* mild cognitive impairment, *SD* standard deviation

*Adjusted for age, gender, race, diabetes, blood pressure (systolic and diastolic levels), and signal strength of OCTA scans

We did not find any significant associations between MMSE and brain imaging findings (MTA, GCA, and WHM), after controlling for covariates (Tables S1–4). We next examined the diagnostic performance of OCTA parameters to detect AD/MCI from controls and compared it with brain MRI (MTA). The values for AUC, sensitivity at 41.5% specificity for the top 3 best parameters, and MTA are presented in Additional file: Table S5. The 3 best parameters to discriminate between AD and MCI vs controls were superficial fractal dimension (AUC, 0.77), superficial vessel density (AUC, 0.72), and deep vessel density (AUC, 0.64; Additional file: Figure S2). The paired comparisons among these 3 parameters were not statistically significant (*P* = 0.141) whereas it was statistically significant compared to MTA scores ≥ 2 (AUC, 0.56; *P* = 0.01) (Fig. S2).

## Discussion

In this cross-sectional study, we evaluated the extent and pattern of retinal microvascular alterations, specifically at the capillary network level, in AD and MCI. We compared three retinal OCTA metrics (VD, FD, and FAZ area) in two capillary plexuses (superficial and deep) in AD, MCI, and controls. Compared with controls, AD participants showed significantly sparser VD in both plexuses whereas MCI participants only showed reduction at the superficial plexus. In terms of FD, AD and MCI participants exhibited a loss of vessel complexity of the SCP when compared with controls. Our study adds further to the concept that there are possible progressive differences in retinal microvascular alterations between AD and MCI; the use of VD in the SCP (together with DCP) may further distinguish between AD (both SCP and DCP affected) and MCI (only SCP is affected) individuals. Taken together with increasing evidence from other research, our current study demonstrates that differences in retinal microvascular changes using OCTA may potentially be used to identify and screen for AD and earlier cognitive phenotypes (i.e., MCI).

### SCP in AD individuals

We showed that AD participants have a sparser VD of the SCP compared with control participants. Our findings support most of the previous OCTA studies in AD participants (Table 4) [12–14, 16], which is in keeping with studies on larger retinal vessels using fundus photographs [3, 40]. It should be noted that three other studies did not observe any differences in the VD of the SCP in AD participants [11, 15, 37]. Furthermore, a study in presymptomatic AD found Aβ+ participants had higher VD on OCTA than Aβ− participants, after accounting for age [41]. OCTA quantification metrics may potentially be affected by several confounders. First, OCTA signal strength quality can affect the VD, where the VD decreased linearly with signal strength [42]. In our study, the scan qualities were extremely high between controls and AD/MCI (9.6 vs 9.8 out of 10) and we adjusted the OCTA signal strength quality. Second, although studies have excluded participants with uncontrolled hypertension, the BP levels can affect the VD [30]. In the current study, we statistically adjusted the BP levels to remove the BP bias. Last, the physiological variability of FAZ can affect the VD [43]. This is mainly dependent on how much FAZ one includes in the analytical regions. In eyes with a larger FAZ, the FAZ would occupy a larger portion of the analytical area, resulting in a lower VD. In the current study, we mitigated the potential measurement bias by manually delineating the FAZ region and masking it from the calculation.

**Table 4:** Optical coherence tomography angiography studies on individuals with Alzheimer’s disease and mild cognitive impairment

Author	Sample	Neurocognitive diagnosis	OCTA imaging	Adjusted for confounders	Projection artifact removed	Corrected for FAZ	Superficial capillary plexus (SCP)	Deep capillary plexus (DCP)	FAZ
Lahme et al. [14]	36 AD and 38 control	NIA-AA	RTVue XR Avanti	No	No	No	Microvascular densities of SCP were significantly lower in AD than controls. Negative correlation between flow density and Fazekas scale.	No significant difference.	No significant difference.
Jiang et al. [44]	12 AD, 19 MCI, and 21 control	NIA-AA	Zeiss Angioplex	No	Yes	No	Fractal dimensions of SCP were significantly lower in AD than controls.	Fractal dimensions of DCP were significantly lower in AD and MCI than controls. Fractal dimensions were positively related to MMSE in MCI patients.	Not available.
Bulut et al. [12]	26 AD and 26 control	Clinically (NIA-AA, DSM-IV)	RTVue XR100-2	No	Not available	No	Microvascular densities of SCP were significantly lower in AD than controls. Correlations were found between MMSE and vascular densities and FAZ.	Not available.	FAZ was significantly enlarged in AD than controls.
Zhang et al. [16]	16 AD/MCI and 16 control	NIA-AA	RTVue XR Avanti	Age-matched	Yes	No	Microvascular densities of SCP were significantly lower in early AD or amnestic type MCI than controls. Positive correlations between vascular densities of SCP and MoCA.	No significant difference.	Not available.
Zabel et al. [11]	27 AD and 27 control	Clinically (NIA-AA, DSM-IV) and radiologically (PET scan)	RTVue XR Avanti	No	Yes	No	No significant difference.	Microvascular densities of DCP were significantly lower in AD than controls.	FAZ was larger in AD than controls.
Yoon et al. [13]	39 AD, 37 MCI, and 133 control	NIA-AA	Cirrus 5000 Angioplex	Yes	Not available	No	Microvascular densities of SCP were significantly lower in AD than controls and AD vs MCI but not between MCI and controls.	Not available.	No significant difference.
den Haan et al. [37]	48 AD and 48 controls	NIA-AA	Cirrus 5000 Angioplex	Yes	Not available	No	No significant difference.	Not available.	No significant difference.
Wu et al. [15]	18 AD, 21 MCI, and 33 control	AD (NINCDS-ADRDA), MCI (Petersen criteria)	RTVue XR Avanti	No	No	No	No significant difference.	Microvascular densities of DCP were significantly lower in AD and MCI than controls.	FAZ was significantly largest in AD, followed by MCI, and lastly controls.
Current study	24 AD, 37 MCI, and 29 control	AD (DSM-IV), MCI (Petersen criteria)	Cirrus 5000 Angioplex	Yes	Yes	Yes	Microvascular densities of SCP were significantly lower in AD and MCI than controls.	Microvascular densities of DCP were significantly lower in AD than controls.	No significant difference.

*AD* Alzheimer’s disease, *DSM-IV* Diagnostic and Statistical Manual of Mental Disorders, *FAZ* foveal avascular zone, *MCI* mild cognitive impairment, *OCTA* optical coherence tomography angiography, *MMSE* Mini-Mental State Examination, *MoCA* Montreal Cognitive Assessment, *NIA-AA* National Institute of Aging-Alzheimer’s Association, *NINCDS-ADRDA* National Institute of Neurological and Communicative Disorders and Stroke and Alzheimer’s Disease and Related Disorders Association

The SCP is responsible for the metabolic supply of the ganglion cell layer [17], where the reduced number of retinal ganglion cells and axons has also been observed in post-mortem AD retinas [7, 8]. Changes in the SCP seen on OCTA further complement the already established retinal OCT structural markers [9]. Using OCT, several studies have reported the reduction of retinal nerve fiber layer thickness and ganglion cell layer thickness (presumably due to loss of retinal ganglion cells and axonal degeneration) in AD patients [9]. It remains unknown the reason for a loss of retinal vessels in AD individuals. One speculation may be due to the loss of neurons or vice versa that was reported in some studies [7, 8]. However, other recent studies did not report a loss of retinal neurons in AD individuals compared to controls [45–48]. Another potential reason for the sparser capillary network in persons with cognitive impairment may be the faulty Aβ clearance from retina. Impaired vascular clearance of Aβ from brain has been implicated in Alzheimer’s disease [49]. Interestingly, Guo et al. showed that targeting Aβ clearance was effective at reducing retinal ganglion cell death in an animal model of glaucoma [50]. Future study would need to perform in vivo OCTA imaging in animal models and correlate with immunocytochemical staining of Aβ.

### SCP in MCI individuals

Discordant results have been reported on the OCTA findings in MCI participants (Table 3). The current and one previous study [16] showed a reduced VD of the SCP in those with MCI whereas other studies did not report any differences [13, 15]. Alteration in the retinal vessels in MCI participants is compatible with studies using in vivo Doppler imaging techniques, where a decrease in retinal blood flow has been demonstrated in both AD and MCI participants [51, 52]. The conflicting results between MCI and control participants may lie with the definition of MCI, which represents a continuum of cognitive decline between “normal aging” and dementia. While the person is still able to carry out their activities of daily living with little or no help from others, a wide range of cognitive impairment is possible in MCI [53]. It is plausible that the change in retinal capillaries may occur only at a more severe stage of MCI or when certain cognitive ability is affected.

### DCP in AD individuals

Four OCTA studies have investigated DCP VD in AD individuals, but there is generally a lack of agreement between studies (Table 4). Two studies [11, 15] showed a significant reduction in VD in AD individuals whereas the others [14, 16] did not observe any differences. Obtaining accurate OCTA metrics from the DCP layer is particularly challenging as it is affected by the physiologic variability of FAZ [18, 19] and projection artifacts [20, 21]. First, previous OCTA studies did not account for the FAZ in the deeper plexus. This is crucial because the FAZ in the deep plexus is considerably larger than superficial plexus [54]. Second, while Zabel et al. removed the projection artifacts in the DCP [11], the rest did not [14–16]. In the current study, we quantified the VD of the DCP without the influence of FAZ and projection artifacts, which hopefully reduced measurement bias. We found a sparser VD of the DCP in AD participants but not in MCI individuals, which suggests the possibility of using the VD of the DCP to discriminate between AD (both SCP and DCP affected) and MCI (only SCP is affected) individuals.

The DCP is important for nutrition of the inner nuclear layer, which comprises bipolar cells, horizontal cells, and amacrine cells [17]. In transgenic AD mouse models, Aβ deposits have been detected in the inner nuclear layer [10]. Microvascular changes of the DCP are complemented by the structural thinning of the inner nuclear layer thickness in AD individuals [55]. Changes in the DCP in AD individuals may present later in the disease stage, but a longitudinal study will be required to confirm this hypothesis.

### Fractal dimension

In addition to capillary loss, we saw a significantly decreased FD of the SCP, which suggests a loss of vessel complexity in the inner retinal macula of those with AD and MCI compared to cognitively normal controls. However, an earlier study [44] observed a significant reduction in the FD in both plexuses in AD and MCI individuals. In contrast, we did not find any difference in the FD of the DCP between the groups. A plausible reason for the disagreement may be related to the vascular arborization pattern of the distinct layers. The SCP is supplied by the central retinal artery and composed of vessels running parallel of the retinal surface, thereby displaying a distinct vascular tree whereas the DCP is supplied by vertical anastomoses from the SCP, presenting as a lobular configuration [17]. Since FD is a measure of vasculature branching pattern complexity, it may be a more relevant biomarker for the SCP than the DCP. Our finding is in keeping with previous publications on larger retinal vessels using fundus photographs, where AD and MCI participants demonstrated a loss of vessel complexity [35].

Our results showed that there was lower fractal dimension in the MCI and AD groups compared with the control group, whereas there was no difference in the fractal dimension between the MCI group and AD group. Pathophysiologically, a lower fractal dimension indicates a loss of vessel complexity, which suggests loss of branch or rarefaction of the retinal vasculature. Similarly, in the brain, a reduction of the small vessels has been observed [56], suggesting that there may be parallel pathological mechanisms at work in the brain and the retina leading to microvascular changes. Taken together, these morphological changes in the retinal microvasculature that are associated with cognitive status confirm that the eye may reveal novel insights into the vascular mechanisms underlying cognitive dysfunctions.

### Perfusion density of large vessels

It should be noted that although VD in SCP was decreased in participants with cognitive impairment, perfusion density of the large retinal vessels remained unchanged. Previous studies have examined retinal vessels from fundus photographs and reported alterations in the venular caliber in participants with AD [3]. Retinal vessels measured from fundus photos are considerably larger in diameter than those obtained from OCTA [4]. Also, the diameters of retinal vessels measured with OCTA are in good agreement with the ground truth as obtained with adaptive optics ophthalmoscope [57]. Therefore, the lack of large vessel changes despite capillary changes suggests that microvascular alterations precede large vessel changes. The capillaries in the SCP may be particularly susceptible to the deleterious effects of neurodegeneration whereas the large retinal vessels may change later in the pathogenesis of AD. This finding would also suggest that the use of OCTA may be more sensitive in detecting changes in AD and MCI participants than fundus camera.

### Foveal avascular zone area

Previous OCTA studies have quantified the FAZ area within the SCP region automatically using the OCTA software and reported conflicting results (Table 4) [11–15, 37]. Some reported a significant enlargement of the FAZ in individuals with AD compared to controls [11, 12, 15], whereas others reported no difference in the FAZ area [13, 14, 37]. In the current study, we did not find any differences in either of the plexuses between groups. Overall, the FAZ area has numerous limitations to serve as a biomarker of cognitive impairment given its physiologic variability, effect of axial length on OCT scan dimensions, and segmentation/measurement limitations [32, 43, 58–60].

### OCTA parameters in relation to cognitive function and brain imaging findings

In the current study, we did not find any significance between OCTA parameters and MMSE or brain imaging findings. While some studies found lower MMSE scores with sparser VD [12, 13, 16], others did not find any association between OCTA parameters and MMSE [11, 14, 37, 44]. Two studies found higher VD in AD and MCI participants with fewer WMH, based on Fazekas scores [14, 37]. Therefore, in our future study, we plan to recruit more participants to achieve enough study power.

### Diagnostic performance of OCTA to detect cognitive impairment

We found that the OCTA parameters were superior to MTA scores for distinguishing between normal and AD/MCI participants. This is in favor of the potential clinical utility of the OCTA as a screening tool for cognitive impairment. Incorporating both components of the retinal microvasculature and retinal ganglion cell axons [9] may further improve the screening performance of OCTA for cognitive impairment. We had previously shown that the structural component of the retina can be extracted from OCTA system with good reliability [61]. Nevertheless, there are many important considerations when determining whether some form of screening for cognitive impairment in older adults is worthwhile [62]; these include the technical challenge of motion artifacts in OCTA scans.

### Strengths and limitations

Strengths of this study include a well-phenotype cohort of AD and MCI individuals who were diagnosed according to internationally accepted criteria, and a standardized study methodology which further improved the validity of the imaging data. As explained above, we accounted for larger retinal vessels, FAZ dimension, and projection artifacts from the analysis. Second, a quarter of our participants were excluded because of poor quality OCTA scans. Such high exclusion is comparable to other OCTA studies (~ 22% OCTA scans were rejected) [13]. Since OCTA is based on motion detection, it is particularly sensitive to the patient’s eye movement. The need for good patient fixation can be challenging in elderly patients with cognitive impairment. Incorporating eye tracking during OCTA scanning can lessen the eye movement [63] but may lead to longer image acquisition time, which in turn result in patient fatigue. In the future, faster scanning speeds would be crucial to overcome the challenge posed by eye motion and improve its usability for everyone, especially in challenging situations [64]. Nonetheless, we performed rigorous quality control on all scans, where all B-scans were checked for misalignment and segmentation errors. Images with segmentation errors, poor signal strength, or eye diseases, which might potentially affect the OCTA measurements, were excluded. This robust data preparation is also evident by the particularly high signal strength among our participants and further corroborates the validity of our results. Third, we adjusted the possible confounding effects of age, gender, race, and the presence of diabetes, and systemic blood pressure levels during multivariate analysis and excluded possible confounding factors, such as eye diseases [29, 30, 39].

Our present study has a few limitations. First, even though there was reduced retinal VD in those with AD and MCI using OCTA, this was only a cross-sectional study. It remains unclear whether the changes of retinal capillary are predictive of cognitive decline. This will be investigated in ongoing follow-up studies. Second, quantification of VD [18] and FAZ [60] can be affected by the effect of OCT magnification. We did not perform ocular biometry and thus were not able to rescale the scan dimensions [18]. Instead, we masked the FAZ from the VD metrics which should have mitigated some of this measurement bias for the VD maps. We acknowledged that the FAZ area is still affected by the individual differences in axial length. Third, the relatively small sample size of those with AD and MCI limits the power for evaluating the OCTA metrics in different types of MCI and AD. Another potential limitation is that we could not confirm the etiological diagnosis of our participants as we did not have cerebrospinal fluid (CSF) or amyloid PET biomarkers in majority of the participants. Lastly, this study was restricted to older adults of Asian ethnicities; therefore, generalizability of our results to persons with young-onset dementia or of non-Asian ethnicities may be limited. Also, it remains unclear how the OCTA relates with magnetic resonance imaging (MRI) markers of cerebrovascular disease markers and amyloid PET and CSF biomarkers.

## Conclusions

Our study shows that compared to cognitively normal controls, eyes of participants with AD demonstrated significantly sparser VD in both plexuses whereas MCI participants only showed reduction of the SCP. Furthermore, we found that AD and MCI participants exhibited a loss of vessel complexity of the SCP when compared with controls. Our findings suggest that the changes at the retinal microvasculature at the capillary level may reflect similar changes in the cerebral vasculature in individuals with neurodegenerative diseases and demonstrate the potential of OCTA for the early screening of cognitively impaired individuals.

## Supplementary Information

**Additional file 1: Supplementary information: Figure S1.** Identification of study participants. Of the 165 participants, we excluded participants who were unable to complete OCTA scanning due to fatigue (*n* = 15), poor scan quality (*n* = 53), and presence of eye diseases (*n* = 7). Among the 90 participants included for the analysis, 24 were Alzheimer’s disease (AD) participants, 37 were mild cognitive impairment (MCI) participants, and 29 were controls. **Figure S2.** Receiver operating characteristic curves and corresponding AUCs for the top 3 parameters for discriminating AD/MCI from controls as compared to MTA scores ≥ 2. FD = fractal dimension; MTA = medial temporal atrophy; and VD = vessel density. **Table S1.** Multivariate analysis of OCTA metrics with MMSE scores **Table S2.** Multivariate analysis of OCTA metrics with presence of medial temporal atrophy (MTA) **Table S3.** Multivariate analysis of OCTA metrics with global cortical atrophy (GCA) **Table S4.** Multivariate analysis of OCTA metrics with white matter hyperintensities (WMH). **Table S5.** Diagnostic performance for cognitive status in decreasing order of the most relevant parameters.

## Data Availability

The datasets used and/or analyzed during the current study are available from the corresponding authors on reasonable request. The dataset(s) supporting the conclusions of this article is (are) included within the article (and its additional file(s)).

## Abbreviations

AD: Alzheimer’s disease
Aβ: β-Amyloid
BP: Blood pressure
DCP: Deep capillary plexus
DSM: Diagnostic and Statistical Manual of Mental Disorders
FAZ: Foveal avascular zone
FD: Fractal dimension
ILM: Inner limiting membrane
IPL: Inner plexiform layer
MCI: Mild cognitive impairment
OCTA: Optical coherence tomography angiography
OPL: Outer plexiform layer
SD: Standard deviation
SCP: Superficial capillary plexus
VD: Vessel density
