# Supplementary material for: Retinal microvasculature dysfunction is associated with Alzheimer’s disease and mild cognitive impairment
Source: Alzheimers Res Ther. 2020 Dec 4;12:161. doi: 10.1186/s13195-020-00724-0 (PMC7718666; doi:10.1186/s13195-020-00724-0)
Supplement: Supplementary file 1 — Additional file 1: Supplementary information: Figure S1. Identification of study participants. Of the 165 participants, we excluded participants who were unable to complete OCTA scanning due to fatigue (n = 15), poor scan quality (n = 53), and presence of eye diseases (n = 7). Among the 90 participants included for the analysis, 24 were Alzheimer’s disease (AD) participants, 37 were mild cognitive impairment (MCI) participants, and 29 were controls. Figure S2. Receiver operating characteristic curves and corresponding AUCs for the top 3 parameters for discriminating AD/MCI from controls as compared to MTA scores ≥ 2. FD = fractal dimension; MTA = medial temporal atrophy; and VD = vessel density. Table S1. Multivariate analysis of OCTA metrics with MMSE scores Table S2. Multivariate analysis of OCTA metrics with presence of medial temporal atrophy (MTA) Table S3. Multivariate analysis of OCTA metrics with global cortical atrophy (GCA) Table S4. Multivariate analysis of OCTA metrics with white matter hyperintensities (WMH). Table S5. Diagnostic performance for cognitive status in decreasing order of the most relevant parameters. [file 13195_2020_724_MOESM1_ESM.pdf]

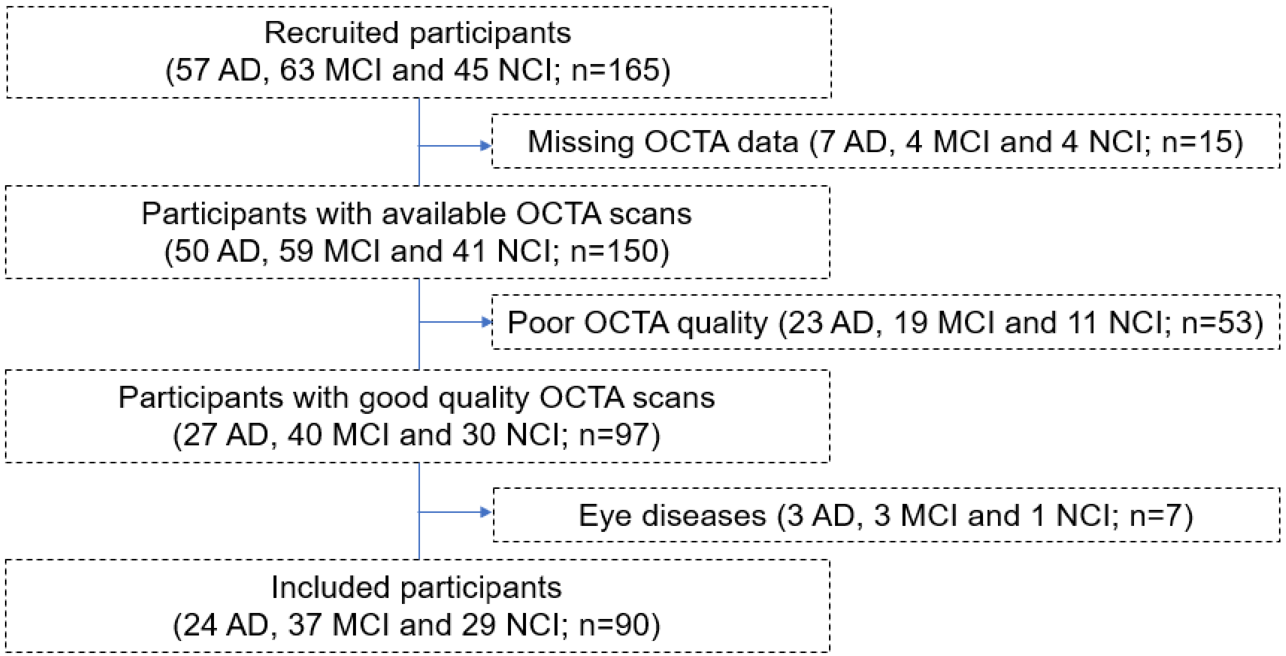

Figure S1: Identification of study participants. Of the 165 participants, we excluded participants who were unable to complete OCTA scanning due to fatigue (n=15), poor scan quality (n=53), and presence of eye diseases (n=7). Among the 90 participants included for the analysis, 24 were Alzheimer's disease (AD) participants, 37 were mild cognitive impairment (MCI) participants, and 29 were controls.

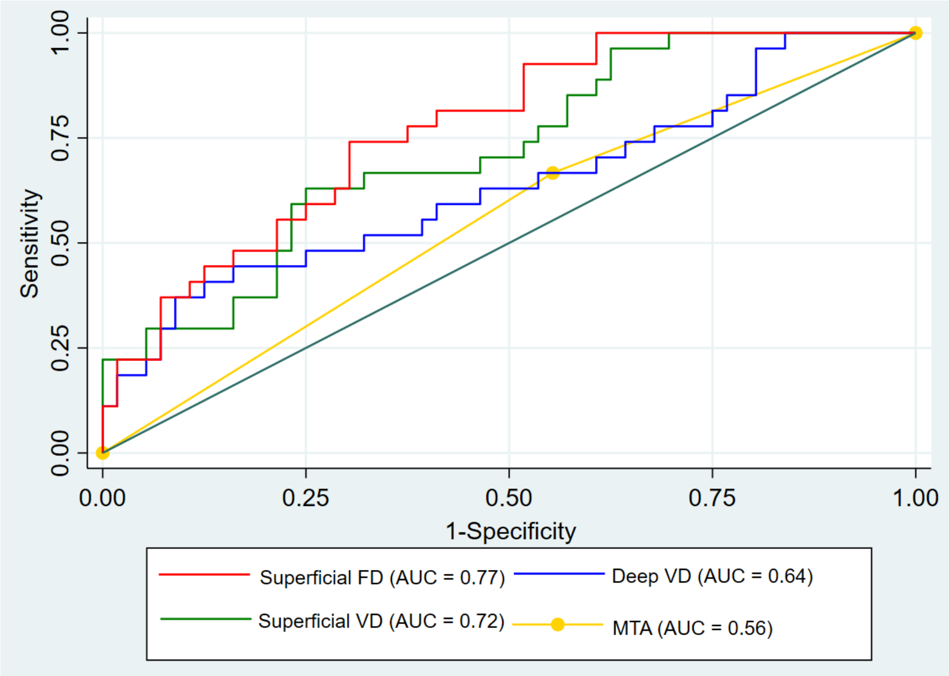

Figure S2: Receiver operating characteristic curves and corresponding AUCs for the top 3 parameters for discriminating AD/MCI from controls as compared to MTA scores $\geq$ 2. FD = fractal dimension; MTA = medial temporal atrophy; and VD = vessel density.

Table S1. Multivariate analysis of OCTA metrics with MMSE scores

OCTA metrics	Superficial capillary plexus			Deep capillary plexus		
	**β**	**95 CI**	**P value**	**β**	**95 CI**	**P value**
Vessel density (mm^{-1})	0.02	-0.04 to 0.04	0.909	0.02	-0.04 to 0.08	0.473
Fractal dimension	0.04	-0.04 to 0.05	0.855	0.01	-0.01 to 0.01	0.105
Perfusion density of large vessels (%)	-0.01	-0.06 to 0.05	0.851	-	-	-
Foveal avascular zone area (mm^2)	-0.04	-0.05 to 0.04	0.988	-0.04	-0.05 to 0.01	0.961

Confidence intervals (CI), mini-mental state exam (MMSE) and standard deviation (SD).

* Adjusted for age, gender, race, diabetes, blood pressure (systolic and diastolic levels) and signal strength of OCTA scans.

Table S2. Multivariate analysis of OCTA metrics with medial temporal atrophy (MTA)

OCTA metrics	Superficial capillary plexus			Deep capillary plexus		
	**β**	**95 CI**	**P value**	**β**	**95 CI**	**P value**
Vessel density (mm^{-1})	0.01	-0.31 to 0.32	0.973	-0.23	-0.71 to 0.24	0.327
Fractal dimension	-0.001	-0.004 to 0.003	0.935	-0.001	-0.005 to 0.003	0.678
Perfusion density of large vessels (%)	0.12	-0.25 to 0.49	0.527	-	-	-
Foveal avascular zone area (mm^2)	-0.02	-0.06 to 0.02	0.307	-0.02	-0.14 to 0.10	0.740

Confidence intervals (CI), medial temporal atrophy (MTA), and standard deviation (SD).

* Adjusted for age, gender, race, diabetes, blood pressure (systolic and diastolic levels) and signal strength of OCTA scans.

Table S3. Multivariate analysis of OCTA metrics with global cortical atrophy (GCA)

OCTA metrics	Superficial capillary plexus			Deep capillary plexus		
	β	95 CI	P value	β	95 CI	P value
Vessel density (mm^{-1})	-0.13	-0.47 to 0.22	0.472	-0.07	-0.58 to 0.43	0.769
Fractal dimension	-0.001	-0.005 to 0.002	0.459	0.001	-0.003 to 0.005	0.633
Perfusion density of large vessels (%)	-0.05	-0.44 to 0.34	0.802	-	-	-
Foveal avascular zone area (mm^2)	-0.02	-0.06 to 0.02	0.275	-0.03	-0.15 to 0.09	0.582

Confidence intervals (CI), global cortical atrophy (GCA), and standard deviation (SD).

* Adjusted for age, gender, race, diabetes, blood pressure (systolic and diastolic levels) and signal strength of OCTA scans.

Table S4. Multivariate analysis of OCTA metrics with white matter hyperintensities (WMH)

OCTA metrics	Superficial capillary plexus			Deep capillary plexus		
	β	95 CI	P value	β	95 CI	P value
Vessel density (mm^{-1})	-0.03	-0.34 to 0.29	0.864	-0.03	-0.50 to 0.45	0.904
Fractal dimension	-0.002	-0.003 to 0.003	0.927	0.001	-0.003 to 0.005	0.519
Perfusion density of large vessels (%)	0.37	-0.35 to 0.38	0.931	-	-	-
Foveal avascular zone area (mm^2)	-0.01	-0.04 to 0.03	0.909	0.01	-0.11 to 0.11	0.972

Confidence intervals (CI), standard deviation (SD) and white matter hyperintensities (WMH).

* Adjusted for age, gender, race, diabetes, blood pressure (systolic and diastolic levels) and signal strength of OCTA scans.

Table S5. Diagnostic performance for cognitive status in decreasing order of the most relevant parameters

**Parameter**	**Area under the Receiver Operating Characteristic Curve (standard error)**	**Sensitivity at 41.5% Specificity**	**P value**
**A) MCI/AD vs Control**			
1. Superficial fractal dimension	0.77 (0.05)	85.7	Reference
2. Superficial vessel density	0.72 (0.06)	64.3	0.358
3. Deep vessel density	0.64 (0.07)	92.9	0.141
4. MTA scores $\geq$ 2	0.56 (0.06)	65.5	**0.010**

Results for sensitivity is expressed as percentages. P value indicates the paired comparisons with the best parameter (reference group).
